# Consistency of a clinical decision support system with molecular tumour board recommendations for tumour sequencing-guided treatment of pancreatic cancer

**DOI:** 10.1016/j.esmogo.2024.100070

**Published:** 2024-06-19

**Authors:** M. Kordes, L. Malgerud, J.-E. Frödin, J. Yachnin, C. Fernandez Moro, S. Ghazi, R. Pozzi Mucelli, N. Kartalis, P. Ghorbani, M. Del Chiaro, V. Wirta, M. Björnstedt, M.G. Liljefors, J.-M. Löhr

**Affiliations:** 1 Department of Clinical Science, Innovation and Technology, Karolinska Institutet, Stockholm; 2 Department of Upper Abdominal Diseases, Karolinska University Hospital, Stockholm; 3 Department of Oncology-Pathology, Karolinska Institut, Stockholm; 4 Phase 1-Unit, Center for Clinical Cancer Studies, Karolinska University Hospital, Stockholm; 5 Department of Laboratory Medicine; 6 Department of Clinical Pathology and Cancer Diagnostics; 7 Department of Radiology Huddinge, Karolinska University Hospital, Stockholm; 8 Department of Science for Life Laboratory, Department of Microbiology, Tumor and Cell Biology, Karolinska Institutet, Stockholm; 9 Department of Genomic Medicine Center Karolinska, Karolinska University Hospital, Stockholm, Sweden

**Keywords:** pancreatic cancer, precision oncology, clinical decision support system, molecular tumour board, cancer genomics, targeted therapies

## Abstract

**Background:**

Pancreatic ductal adenocarcinoma (PDACs) can have actionable genomic alterations at varying frequencies. Harnessing tumour profiling for its treatment is an intricate procedure because the matching of aberrations with therapeutic opportunities is increasingly complex. We evaluated a clinical decision support system (CDSS) that can be used to rationalise this process.

**Methods:**

Patients with advanced PDAC were enrolled in a prospective observational study to assess a CDSS, MH Guide. Sequencing data were analysed with the CDSS, and a study-specific molecular tumour board (MTB) assessed reported actionabilities, ineffective drugs, and excess toxicity warnings. We carried out a *post hoc* analysis of each patient’s treatment within the purview of their medical team and compared it with CDSS and MTB statements.

**Results:**

We included 39 patients in the study, 31 had complete CDSS reports and 28 were discussed at the MTB. The CDSS made 80 treatment suggestions based on 61 actionable variants. It highlighted 14 inefficacy marker–drug pairs based on 7 individual markers and flagged a total of 15 individual markers of increased risks of drug-associated toxicity for 28 different cancer treatments. The study-specific MTB endorsed molecularly informed therapeutic options in 21 cases, but there was no exclusion of any drugs based on inefficacy or toxicity markers. The overall evidence underpinning assertions of actionability was weak. After the end of the study, eight patients received targeted treatment without signs of response or clinical benefit.

**Conclusion:**

The oncology CDSS MH Guide can be deployed along the care path of patients with advanced PDAC but a critical review of assertions from it is warranted.

## Highlights

• Successful deployment of a CDSS in an extensively pretreated population with pancreatic cancer.
• Critical evaluation of automated assertions of actionability, markers of ineffectiveness, and toxicity.
• Survey of the implementation of CDSS assertions and their appreciation by an MTB in clinical routine.

## Introduction

Across a broad spectrum of solid malignancies, treatment directed against specific genomic alterations has been shown to generate clinical benefit for patients.^1^ Comprehensive genomic testing of the tumour is therefore increasingly integrated into the oncology workflow to inform individual treatment decisions guided by the presence of actionable genomic targets in addition to the histologic tumour type.^2^ For patients with advanced disease, especially if no standard of care exists, this approach can direct enrolment into biomarker-selected clinical trials or guide off-label repurposing of drugs registered for other indications.

A crucial step in this process is the accurate interpretation of genomic test results to link detected variants with specific therapeutic options. While this task might seem straightforward, it presents in many cases a formidable challenge where clinicians are required to manually review the scientific evidence to identify and evaluate potential treatment options related to an alteration. As the number of identified drug biomarkers and the available information on the clinical efficacy of molecularly informed therapies are constantly expanding, this process becomes increasingly difficult and likely to suffer from inconsistencies. In addition, medical teams need to consider other clinically relevant genomic events, including germline pathogenic variants and pharmacogenomic markers indicating inefficacy or an increased risk of drug toxicity. Although many medical centres have established multidisciplinary molecular tumour boards (MTBs) to improve the decision-making process, a lack of standardisation can result in different assessments of the clinical relevance of the same genomic alteration across groups of experts.^3^^,^^4^

As one answer to these challenges, expert-curated knowledge bases of predictive genomic biomarker information have been developed to provide the best available evidence for clinical decisions.^5^, ^6^, ^7^ Integrated clinical decision support systems (CDSSs) can harness this information and combine it with automated processing and interpretation of sequencing data before producing a structured report of potential therapeutic options for a specific patient case.^8^^,^^9^ These reports can then be used directly by treating clinicians, or they can support evaluation by an expert panel.

Among solid malignancies, pancreatic ductal adenocarcinoma (PDAC) has a particularly dire prognosis. Most patients present with metastatic disease at diagnosis and 5-year survival rates as low as 1%-2% have been reported in this group.^10^ These patients are not eligible for surgical resection of the tumour and cytotoxic chemotherapy remains the standard of care. However, even with the most effective drug combinations, the median progression-free survival hardly exceeds 6 months and the median overall survival is <1 year.^11^^,^^12^

The tumour genome of most patients with PDAC harbours somatic variants in a few frequently mutated genes, including *KRAS*, *TP53*, *CDKN2A*, and *SMAD4*. At the same time, variants and copy number variations in a broad spectrum of other genes occur at a lower frequency and often involve important oncogenic mechanisms that represent potential therapeutic targets.^13^^,^^14^ Consistent with this observation, subpopulations of patients based on genomic variants have been recognised to benefit from various molecularly targeted therapies.^15^, ^16^, ^17^, ^18^ However, prospective clinical trials that covered a spectrum of potentially actionable molecular targets resulted in few patients receiving any molecularly informed therapies.^19^^,^^20^

In a previous retrospective study of archival material, we demonstrated that a proprietary oncology CDSS, MH Guide (previously branded as Treatment Map, Molecular Health), could be used to systematically analyse PDAC sequencing data.^21^ Based on >50 databases, the CDSS rapidly interprets results and generates a report of potential molecularly informed therapies as well as potential resistance mechanisms and toxicity markers.^22^^,^^23^ Here, we report the results and broader insights from the implementation of this system in the care path of patients with PDAC in a prospective observational study.

## Patients and methods

### Patients

Patients with locally advanced, metastatic, or recurrent PDAC were identified at the gastrointestinal oncology clinic of the Oncology Department at Karolinska University Hospital or enrolled in the trial after referral to our hospital. Patients who had received first-line systemic therapy for advanced disease, and who would qualify for second-line treatment according to investigator evaluation, were eligible for enrolment in the trial. We informed patients about the genomic information that can be obtained with tumour DNA sequencing, including the possibility of accidental germline findings and their implications. Subsequent treatment decisions considering genomics results generated by this study were not regulated by the study protocol. All treatment decisions were independently made by the patient’s medical team and were not determined by CDSS recommendations or MTB statements. The study was approved by the regional ethical review authority (Regionala Etikprövningsnämnden i Stockholm; Dnr 2015/1732-31; Dnr 2016/1106-32; Dnr 2016/2234-32; and Dnr 2017/2033-32). We obtained written informed consent from all patients before they participated in the study. The retrospective *post hoc* assessment of treatment, including targeted therapies, after participation in the study was conducted under a separate waiver from the regional ethical review authority (Dnr 2015/2185-31/4). The study was conducted in accordance with the Declaration of Helsinki. This study is registered at ClinicalTrials.gov with the registration number NCT02767700.

### Tissue processing and DNA extraction

Archival formalin-fixed paraffin-embedded tissue biopsies or surgical specimens were obtained from the Department of Pathology or acquired from the respective pathology provider if the patient was referred from an outside hospital. Haematoxylin–eosin-stained sections were reviewed by study pathologists to validate the diagnosis and determine the tumour content of the sample. We used the standardised workflow of the Department of Pathology for tissue processing, DNA extraction, and quality control.

### Tumour tissue DNA sequencing

DNA target enrichment was carried out manually using optimised protocols (e.g. prolonged hybridisation times, optimised PCR cycles, and washing steps) for an Agilent SureSelect XT custom-designed 620 gene panel (Supplementary Methods, available at https://doi.org/10.1016/j.esmogo.2024.100070). Samples were sequenced with a HiSeq 2500 (Illumina) sequencer in rapid-run mode with paired-end 2 × 100 bp reads. Results were demultiplexed and FASTQ files were generated using Casava 1.8.4 (Illumina). The average fragment length was 200-400 bp. The average coverage achieved was >100×. After quality control, encrypted and pseudonymised FASTQ files were transferred to Molecular Health via a virtual private network gateway for further analysis.

### CDSS data analysis, annotation, and reporting

Genomic data were analysed using the proprietary CDSS MH Guide version 3.0 (Molecular Health). MH Guide is regulated as a CE-marked medicinal product class I. The CDSS processes raw sequencing data from an individual patient using basic demographic and clinical parameters. In the first step, the genome analysis pipeline aligns the sequencing data with ancestry-specific reference genomes. The generated BAM files are then processed through the respective algorithm for variant calling which can detect gene fusion, insertions–deletions (indels), and single-nucleotide variants (SNVs).^24^ Tumour and germline-specific genomic alterations are then mapped to reference proteins using Ensembl database homo_sapiens_core and UniProt. Genome analysis was followed by evidence mining and clinical interpretation using proprietary algorithms (Supplementary Methods, available at https://doi.org/10.1016/j.esmogo.2024.100070). The CDSS creates a report with a hierarchical list of actionable genomic biomarkers and matched therapies in three categories: potentially effective therapies, potentially ineffective therapies, and therapies with an increased risk of adverse reactions. Assertions are ranked based on a proprietary meta-analysis. Each recommendation is, according to the evidence underpinning its soundness, assigned one of three validity levels: ‘Clinical—Approved’ (grade 3) if a companion diagnostic test for this biomarker has been approved by a regulatory agency to predict a specific effect of the drug (i.e. response, resistance, or toxicity) in the tumour type; ‘Clinical’ (grade 2) if no disease-specific companion test has been approved but this biomarker has been observed in patients to predict a specific effect of the drug; and ‘preclinical’ (grade 1) if the biomarker has been observed either (i) in preclinical contexts or (ii) predicted by a computational method and/or expert opinion to predict a specific effect of the drug.

### Re-evaluation of CDSS assertions using the ESCAT

References listed by the CDSS as the basis of assertions of actionability were manually reviewed. We then assigned the asserted molecularly matched therapies a level of evidence according to the ESMO Scale for Clinical Actionability of Molecular Targets (ESCAT) to emulate the evaluation of the evidence available to the CDSS at the time of the study.^25^

### Study-specific MTB

The study-specific MTB comprised a physician trained and certified by the CDSS provider to use the MH Guide, a clinical oncologist, a pathologist, and a tumour biologist with expertise in pancreatic cancer. The MTB could co-opt other specialists to discuss cases requiring additional expertise in other areas, for example, geneticists or nurse coordinators. The MTB met *ad hoc* to discuss study cases based on available results, but at least every other month between April 2017 and March 2018. For each case, the MTB considered the CDSS assertions together with available clinical information and other available molecular analyses. For patients who had tumours with potentially actionable variants, MTB statements could be (i) continued treatment with cytotoxic chemotherapy, (ii) enrolment in a clinical trial, (iii) a statement of support for off-label treatment with a registered drug, (iv) participation in a compassionate use or expanded access program, or (v) best supportive care. The minimum requirement to endorse a CDSS-recommended therapy was that the CDSS assertion provided a biologically sound rationale.

### Statistical analysis

Absolute values, proportions, medians, means, ranges, and other descriptive statistics were reported as appropriate. Analyses and data visualisations were carried out with SPSS, version 25 (IBM, Inc., New York, NY) and Office suite version 16.83 (Microsoft, Redmond, WA).

## Results

### Patient characteristics

**Figure 1:**
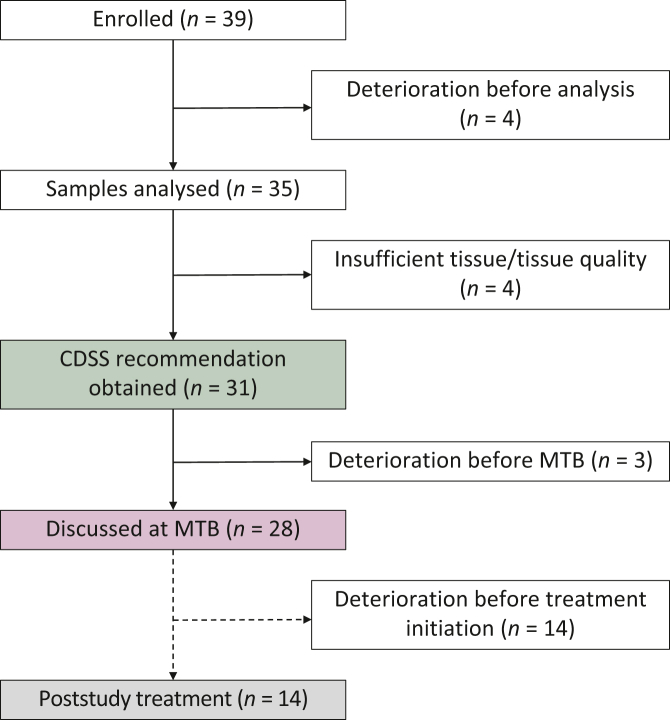
**Flow diagram of patient inclusion and recommendation delineation.** Distribution of individual patients across the different steps of the study. No MH Guide clinical decision support system (CDSS) recommendations and molecular tumour board (MTB) statements were obtained from patients who deteriorated clinically or for whom no sequencing data could be obtained (solid lines). A *post hoc* analysis of the implementation of poststudy treatment of patients for whom CDSS recommendations and MTB statements were available (dashed lines).

Between February and December 2017, 39 patients were enrolled in the study and a CDSS report was obtained in 31 cases (Figure 1). No CDSS report could be obtained for eight patients either due to rapid deterioration of the performance status (*n* = 4) or due to disease progression and technical limitations to carrying out tumour sequencing with the available archival tumour tissue (*n* = 4). Three more patients deteriorated before sequencing results and CDSS recommendations could be discussed at the MTB. For the remaining 28 patients whose cases were evaluated at the MTB, the median time from study enrolment to MTB was 48 days (interquartile range 35.8-70.5 days).

The study cohort was relatively well-balanced between women and men, and slightly skewed towards a younger age range (Table 1). Almost all patients had a very good performance status despite extensive previous treatment. A substantial proportion of the cohort had undergone primary tumour resection (*n* = 17) and adjuvant treatment (*n* = 12), and almost half of all patients (*n* = 19) had received two or more lines of systemic treatment for advanced, metastatic, or recurrent disease. Consequently, sequencing was largely carried out on tissue samples from the primary tumour (*n* = 21). The remainder of the analyses were carried out on core biopsy material from liver metastases (*n* = 15); sequencing of both primary tumour and metastatic tissue was carried out in one patient.

**Table 1:** Patient characteristics

Characteristic	All patients (*n* = 39)	MTB patients (*n =* 28)
Age, years, median (range)	69.2 (48-76.9)	68.7 (50.8-76.9)
Sex, *n* (%)		
Female	17 (43.6)	10 (35.7)
Male	22 (56.4)	18 (64.3)
ECOG, *n* (%)		
0	21 (53.8)	17 (60.7)
1	17 (43.6)	10 (35.7)
2	1 (2.6)	1 (3.6)
Tumour location, *n* (%)		
Head	21 (53.8)	14 (50)
Body	12 (30.8)	9 (32.1)
Tail	4 (10.3)	4 (14.3)
Periampullary	2 (5.1)	1 (3.6)
Metastatic sites^a^, *n* (%)		
None	21 (53.8)	15 (53.6)
Liver	12 (30.8)	9 (32.1)
Lung	2 (5.1)	1 (3.6)
Peritoneum	3 (7.7)	2 (7.1)
Nonregional lymph nodes	1 (2.6)	0 (0)
Other	1 (2.6)	1 (3.6)
Histopathology^b^, *n* (%)		
Pancreatic ductal adenocarcinoma	36 (92.3)	26 (92.9)
Adenosquamous carcinoma	1 (2.6)	1 (3.6)
Mucinous adenocarcinoma	1 (2.6)	—
Adenocarcinoma NOS	1 (2.6)	1 (3.6)
Previous treatment, *n* (%)		
Surgery	17 (43.6)	15 (53.6)
Preoperative chemotherapy	3 (7.7)	3 (10.7)
Adjuvant chemotherapy	12 (30.8)	11 (28.2)
Two or more lines of chemotherapy for advanced disease	19 (48.7)	15 (53.6)
Tissue sample^b^, *n* (%)		
Primary tumour	21	17
Metastasis	15	12

ECOG, Eastern Cooperative Oncology Group; MTB, molecular tumour board; NOS, not otherwise specified.

a Metastatic sites at diagnosis.

b Two separate analyses of tissue from the primary tumour and a metastasis were carried out in one case.

### Tumour tissue sequencing outcomes

**Figure 2:**
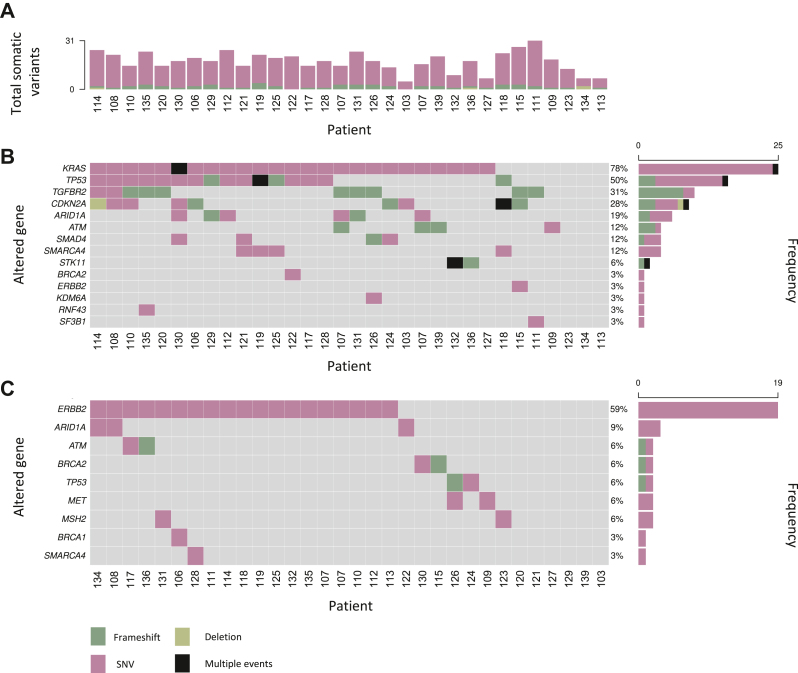
**Summary of detected variants in tumour samples with available clinical decision support system reports.** Columns represent individual samples (*n* = 32). Two independent samples from one individual (patient no. 107) were included. (A) Total number of somatic variants across 620 analysed genes. (B) Oncoplot of somatic variants in 14 selected pancreatic cancer-relevant genes. (C) Oncoplot of germline variants in nine selected genes with hereditary and/or clinical implications for pancreatic ductal adenocarcinoma. SNV, single-nucleotide variant.

The CDSS reported a total of 1077 variants across 422 genes, of which 567 variants in 209 genes were classified as somatic (Supplementary Table S1, available at https://doi.org/10.1016/j.esmogo.2024.100070). Somatic variants with a variant allele frequency ≥10% deemed clinically relevant by the CDSS were found in all 32 analysed samples from 31 individual patients, with a median of 18 variants (interquartile range 15-22 variants) per sample (Figure 2A). Among the 10 most frequently mutated genes were 4 with known implications in pancreatic cancer tumour biology: *KRAS* (*n* = 15; 78%), *TP53* (*n* = 16; 50%), *TGFBR2* (*n* = 10; 31%), and *CDKN2A* (*n* = 9; 28%). Variants in *ARID1A* (*n* = 6; 19%); *ATM* (*n* = 4; 13%) *SMAD4* (*n* = 4; 13%), *SMARCA4* (*n* = 4; 13%), and *STK11* (*n* = 2; 6%), other genes commonly associated with pancreatic cancer, occurred at lower frequencies, and aberrations in some other pancreatic cancer-relevant genes were detected in individual samples (Figure 2B). Across this selected set of genes, SNVs (*n* = 65) and frameshift variants (*n* = 23) clustered distinctly with certain genes.

In addition to somatic alterations, we observed a total of 510 germline variants across 213 genes. Germline variants were reported in all 31 patients with a CDSS report with a median of 16 variants (interquartile range 12.5-19 variants) per sample (Supplementary Table S1, available at https://doi.org/10.1016/j.esmogo.2024.100070). The CDSS recognised eight frameshift (*n* = 7) and deletion (*n* = 1) germline events in this patient cohort including a pathogenic *BRCA*2 K434fs variant that was already known to affect the patient. In a selected set of genes with potential implications for hereditary PDAC, SNVs in *ERBB2* (*n* = 17; 55%) were the most frequently reported variants; variants in *ARID1A* (*n* = 3; 10%), *ATM* (*n* = 2; 7%), *BRCA2* (*n* = 2; 7%); *TP53* (*n* = 2; 7%), *MET* (*n* = 2; 7%), *MSH2* (*n* = 2; 7%), *BRCA1* (*n* = 1; 0.3%), and *SMARCA4* (*n* = 1; 0.3%) were less common (Figure 2C).

### CDSS treatment recommendations

**Figure 3:**
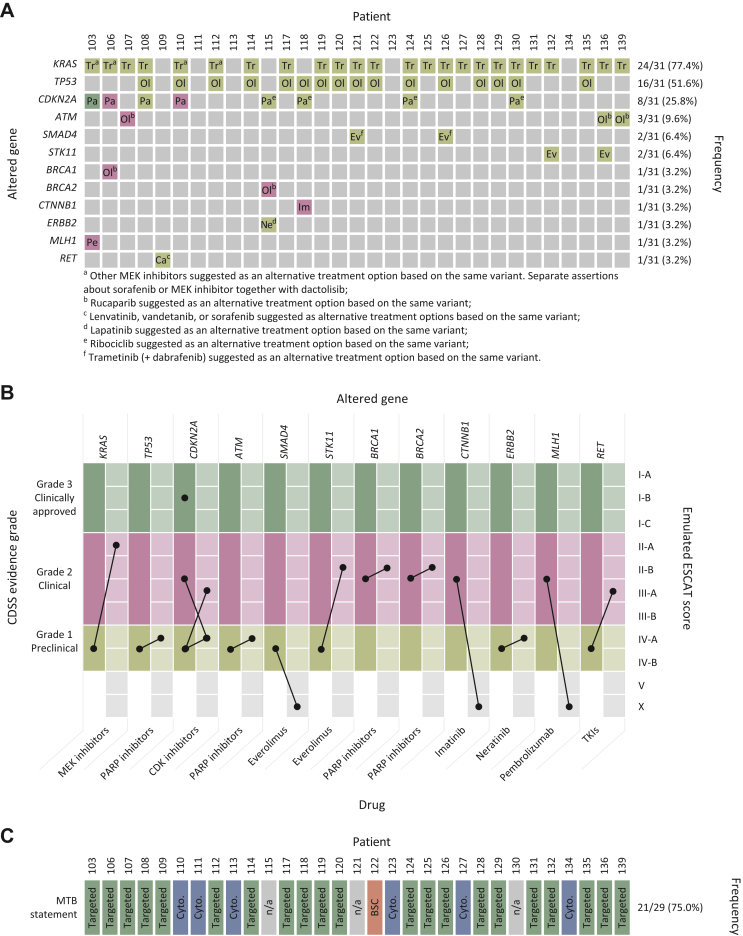
**Molecularly informed clinical decision support system (CDSS) recommendations of potentially active drugs and molecular tumour board (MTB) evaluation.** (A) CDSS recommendations of potentially active drugs. Columns represent individual patients. Rows represent the gene affected by a predictive alteration. Field colours indicate the strength of evidence according to the CDSS: olive, grade 1; burgundy, grade 2; green, grade 3. Ca, cabozantinib; Ev, everolimus; Ne, neratinib; Ol, olaparib; Pa, palbociclib; Pe, pembrolizumab; Tr, trametinib. (B) Stated evidence grade for targeted therapies by gene and drug (left columns) and emulated ESMO Scale for Clinical Actionability of Molecular Targets (ESCAT) scores (right columns). Bars indicate individual assertions. (C) MTB statement on the CDSS report. BSC, ‘Best supportive care recommended’; Cyto, ‘Cytotoxic chemotherapy recommended’; n/a, not applicable; Targeted, ‘Variant-guided options might be considered’. CDK, cyclin-dependent kinase; TKI, tyrosine kinase inhibitor; MEK, mitogen-activated protein kinase kinase; PARP, poly-ADP ribose polymerase.

Among all 31 analysed cases, the CDSS made 80 treatment suggestions based on 61 potentially actionable aberrations (Figure 3A; Supplementary Table S2, available at https://doi.org/10.1016/j.esmogo.2024.100070). The majority of CDSS treatment recommendations were based on variants in *KRAS*, *TP*53, and *CDKN2A*. However, the overall strength of recommendations according to the CDSS classification based on *KRAS* (*n* = 24) and *TP53* (*n* = 16) variants was only level 1. Higher level 2 and 3 evidence strength was assigned to assertions based on variants in *CDKN2A*, *ATM*, *BRCA1*, *BRCA2*, *CTNNB1*, and *MLH1*. Notably, the evidence strength for CDK inhibitors based on *CDKN2A* variants varied across patients between levels 3 (*n* = 1), 2 (*n* = 2), and 1 (*n* = 4). Recommendations based on similar *ATM* variants were also given discordant validity levels (level 1, *n =* 2; level 2, *n* = 1).

When we retrospectively reclassified the CDSS assertions of actionability according to the ESCAT, only the evaluation of the provided references for *TP53*, *ATM*, *BRCA1*, *BRCA2*, and *ERBB2* remained largely unchanged (Figure 3B, Supplementary Table S3, available at https://doi.org/10.1016/j.esmogo.2024.100070). The use of MEK inhibitors for *KRAS*-mutant PDAC was hiked from ‘preclinical’ in the proprietary evaluation to ESCAT tier II-A based on a retrospective analysis of phase I studies.^26^ Similarly, assertions for mutant *STK11* and *RET* were raised from ‘preclinical’ to ESCAT tier II/III based on referenced clinical information.^27^^,^^28^ More concerningly, however, the references given by the system to underpin the use of everolimus for *SMAD4*-, imatinib for *CTNNB1*-, and pembrolizumab for *MLH1*-aberrant disease were in our opinion not supporting the assertion. The references linked to the highest grade of evidence by the CDSS were not relevant for the evaluation of *CDKN2A* and we did not assign an ESCAT tier for this case; inconsistencies in referencing clinical evidence led to the reclassification of the evidence grade in either direction in the remaining cases.

#### Markers of inefficacy

The CDSS reported inefficacy markers for cancer drugs 39 times, and 27 of 31 patients had at least one marker. In total, there were 14 individual inefficacy marker–cancer drug pairs based on 7 individual markers (Supplementary Table S4, available at https://doi.org/10.1016/j.esmogo.2024.100070). These markers comprised a mix of two different germline variants in *CYP2D6* (*n* = 9), four different somatic variants in *KRAS* (*n* = 29), and one variant in *CTNNB1* (*n* = 1). The clinically most relevant alerts for patients with pancreatic cancer were raised for drugs targeting EGFR, the RAS–RAF–MEK pathway, and the phosphatidylinositol-3-kinase–protein kinase B–mammalian target of rapamycin (PI3K–AKT–mTOR) signalling pathway based on *KRAS* codon 12 variants. However, recommendations based on similar genotypes did not result in the same inefficacy alert in all cases. The validity strength assigned to all recommendations was level 2.

#### Germline toxicity markers

Heterozygous germline variants with increased risks of drug-associated toxicity were identified in 30 of 31 patients with a CDSS report; no homozygous variants were detected. A total of 15 individual markers associated with 28 different cancer therapies were reported, including several drugs commonly used to treat PDAC (Supplementary Table S5, available at https://doi.org/10.1016/j.esmogo.2024.100070). The *XRCC1* R399Q polymorphisms (*n* = 20), potentially increasing toxicity associated with platinum agents, were the variants that affected most patients across the cohort. Other alerts regarding gemcitabine (*CDA* K27Q; *n* = 5), fluoropyrimidines (*DPYD* S534N; *n* = 1, *UMPS* G213A; *n* = 1), and irinotecan (*UGT1A* G7IR; *n* = 1) were less frequent. The CDSS assigned the validity level 2 to these PDAC-relevant warnings.

### MTB-endorsed strategies

The study-specific MTB reviewed CDSS reports from 28 patients. Despite the overall low strength of the evidence supporting most suggestions of potentially active drugs, the MTB agreed that in 21 of 28 cases, the CDSS recommendation was sufficiently sound so that variant-guided options might be considered, if warranted by limited alternative treatment options (Figure 3C). In all of these cases, the MTB issued bulk statements supporting consideration of all suggested therapies. None of the patients could be matched to an ongoing clinical trial or expanded access program. In seven cases, the MTB recommended the use of cytotoxic chemotherapy or best supportive care. In the majority of these cases (*n* = 4), the CDSS had not provided any variant-guided treatment recommendation, while the MTB considered other clinical evidence to outweigh the genomic evidence supporting a molecularly informed approach in the remaining three cases. The MTB did not endorse avoiding any drugs based on any of the inefficacy or toxicity alerts raised by the CDSS.

### Poststudy choice of treatment

**Figure 4:**
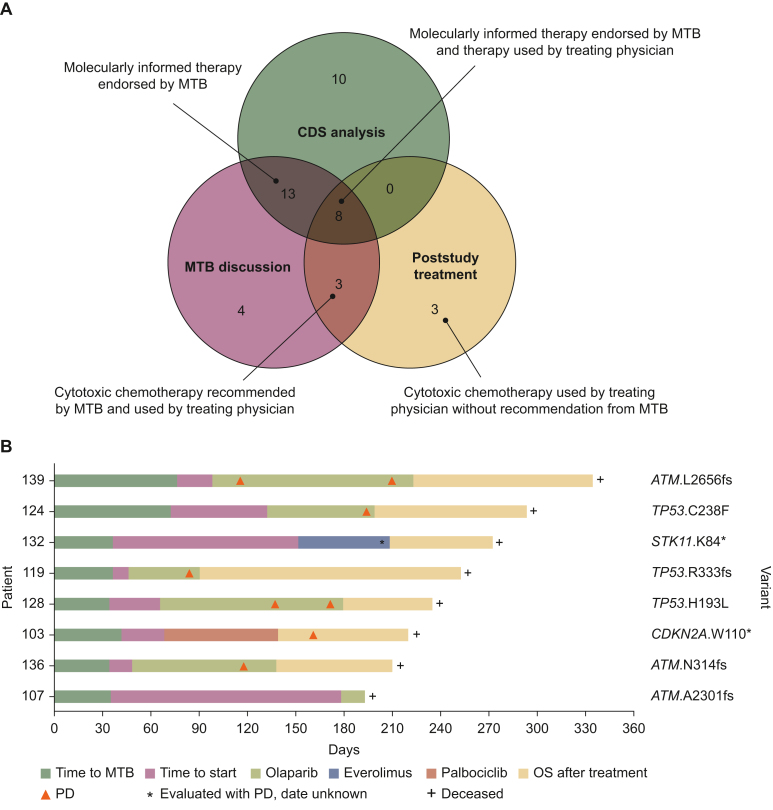
***Post hoc* analysis of targeted therapies after the study.** (A) Modified Venn diagram of clinical decision support system (CDSS) treatment recommendations, molecular tumour board (MTB) statements, and poststudy treatments. Green circle, available CDSS recommendations (*n* = 31); burgundy circle, MTB statements (*n* = 28); yellow circle, patients who received poststudy treatment per *post hoc* analysis (*n =* 14). (B) Swimmer plot of patients who received molecularly matched therapies after the study (*n* = 8). OS, overall survival; PD, progressive disease.

We carried out a *post hoc* analysis of the study participants’ clinical care path to assess whether CDSS recommendations or MTB statements were reflected in the treatment they received in routine care (Figure 4A). Actionable alterations reported by the CDSS converged with MTB evaluation of the report and poststudy treatment in eight cases (i.e. 26% of patients with an available report and 20% of all study participants). Three patients received cytotoxic chemotherapy in line with a CDSS assertion and MTB statement. In three other cases, the patients’ medical teams decided to use cytotoxic chemotherapy, although the CDSS and MTB had considered targeted strategies.

Among the eight patients treated with a targeted therapy, six received olaparib. Of these, three patients had *ATM* variants and three had *TP53* variants (Figure 4B). The remaining two patients received everolimus and palbociclib based on an *STK11* and a *CDKN2A* variant, respectively. The median time to treatment failure was 69 days (range 15-125 days). Seven patients had progressive disease at the first radiological evaluation and one patient died of disease progression shortly after treatment initiation.

## Discussion

Resistance to cytotoxic chemotherapy is one of the hallmarks of PDAC.^29^ Precision therapies might help to overcome this important clinical problem, but actionable genomic aberrations occur across a broad range of genes with a low prevalence of most actionable variants.^30^ Strategies to systematically evaluate the available evidence for molecularly informed treatments are therefore crucial to broadly implement and scale precision oncology for these patients. The use of a CDSS can address this need by reducing the required manual work to evaluate relevant variants and by standardising their interpretation.

In this prospective observational trial, we applied the CDSS MH Guide to PDAC sequencing data and reviewed the output in a study-specific MTB to assess the clinical relevance for individual patients. Given that the study was conducted in 2017/2018 and that the knowledge that can be leveraged to evaluate pancreatic cancer genomic profiles has expended since, some of the assertions made in this study no longer reflect the best available evidence. This is particularly important for assertions based on *KRAS*, *TP53*, and *CDKN2A* variants, all of which can very likely not be acted upon with the suggested approaches.^31^, ^32^, ^33^ Similarly, the CDSS is not evaluated in a current up-to-date iteration, limiting conclusions regarding its clinical utility today. However, we believe that broader lessons for the use of CDSS and for conducting CDSS-supported MTBs can be learned from this study.

Although the main findings of this study are (i) a high rate of successful output from the CDSS within a reasonable time frame in an extensively pretreated population with PDAC, (ii) the identification of potentially relevant actionable variants and markers of inefficacy or of toxicity in most analysed samples, and (iii) a high consensus between CDSS recommendations based on actionable variants and the MTB evaluation, the study exemplifies important general considerations in all three domains.

In the first domain, the observed frequency of *KRAS* and *SMAD4* variants deviates from commonly accepted rates, which raises questions about the cohort’s representativeness. Participation in precision oncology trials is prone to selection bias related to various factors,^34^ and we cannot exclude that our cohort of extensively pretreated patients has an overrepresentation of patients with a different tumour biology than the general patient population. However, the demographics and survival data of the entire cohort do not point to any clinical differences from the accepted presentation of pancreatic cancer. Given the small sample size, the somewhat lower frequency of certain common alterations might be a coincidence. When compared with other PDAC studies using tumour sequencing, we achieved a high technical success rate with interpretable results from archival material.^19^^,^^35^

In the second domain, the inconsistencies between similar molecular cases raise critical questions about the internal validity of the system. Unfortunately, it is not evident why the proprietary algorithm made discordant recommendations and this inconsistent variant interpretation jeopardises one of the core advantages of a CDSS, the scalability of the precision oncology approach. This is compounded by sometimes deficient classification of evidence, for example, assigning the level ‘preclinical’ to clinical data and vice versa. A possible explanation for this could be that the algorithm uses too broad biomarker definitions, for example, entire genes or pathways that do not capture the complexity of individual aberrations.

In addition to internal validity issues, our study highlights the need to assure the external validity of assertions. In our case, the CDSS did not consider crucial evidence available at the time of the study (e.g. the suggestion to use pembrolizumab based on the *MLH1* I219V variant, which is known to be a benign polymorphism).^36^^,^^37^ By contrast, some actionabilities were linked to references that were not relevant for the marker–drug pair in question.

Together, these irregularities increased the rate of patients with actionable aberrations which was higher than in larger cohorts that relied on MTB discussions without any CDSS support to identify molecular targets.^20^^,^^38^ To safeguard the correct interpretation of variants with a CDSS in a clinical context, we suggest a combination of routines and technical approaches (Table 2).

**Table 2:** General considerations for CDSS and CDSS-supported MTBs

Issue	Description	Potential solution
Internal validity	• CDSS reports inconsistent across similar molecular cases	• Manual review of assertions • Transparent reporting of criteria and used evidence • Open-source algorithms
External validity	• CDSS does not consider relevant evidence	• Transparent reporting of criteria and used evidence • Manual validation with public sources • Variant-based assertions instead of broad biomarker definitions • Expert/MTB review
MTB role	• Cognitive bias towards agreeing with assertions • Overoptimism with respect to targeted therapies	• Use of established frameworks to evaluate molecular aberrations • Sufficient expertise • Structured workflow/discussion • Routines for evaluation of automated reporting

CDSS, clinical decision support system; MTB, molecular tumour board.

In the third domain, the study-specific MTB endorsed assertions of actionability with mixed and low-grade evidence. This tendency might be reinforced by a cognitive bias towards endorsing CDSS assertions instead of opposing to them, especially if there is an additional incentive to agree with them in the context of limited other treatment options. While we did not systematically evaluate the individual decision processes in the MTB for each case, these dynamics can lead to an overoptimism with regard to targeted therapies which sharply contrasts with the lack of efficacy that we observed in our *post hoc* analysis. To hedge against bias in the MTB, a combination of expertise and routines should be considered. With regard to markers of drug sensitivity/toxicity, the MTB reviewed all assertions but did not consider any of these solid enough to drive the omission of any treatment.

### Conclusion

While we successfully deployed the CDSS MH Guide along the care path of patients with PDAC, the interpretation of molecular aberrations could not be reliably automated and critical appreciation by human experts remains crucial. Precision treatment based on some of the asserted actionabilities was not associated with responsiveness or clinical benefit.
