# Supplementary material for: Consistency of a clinical decision support system with molecular tumour board recommendations for tumour sequencing-guided treatment of pancreatic cancer
Source: ESMO Gastrointest Oncol. 2024 Jun 19;5:100070. doi: 10.1016/j.esmogo.2024.100070 (PMC12836574; doi:10.1016/j.esmogo.2024.100070)
Supplement: Supplementary Methods [file mmc1.pdf]

Consistency of a clinical decision support system with molecular tumour board recommendations for tumour sequencing-guided treatment of pancreatic cancer: A prospective observational study

Supplemental Methods

1. Description of the MH Guide software....1
Tumour genome analysis....1
Evidence mining....2
Clinical Interpretation:....2
2. MH Guide Biomarker validity scores....3
3. List of targeted genes....4
4. References....6

1. Description of the MH Guide software

Tumour genome analysis

The first major step is the genome data analysis. Here, the clinical decision support system detects genetic alterations in a patient's tumour, based on an analysis of their raw sequencing data. Targeted panel sequencing information is analysed in a non-paired fashion and does not include a comparison to the patient's germline reference. The genome analysis pipeline uses a defined set of quality-controlled, standard analytical applications and reference resource databases that are connected in a controlled workflow. The tools of the pipeline were selected by evaluating sensitivity and precision using synthetic patient data with known variants.[1,2]

The genome analysis pipeline takes raw sequence data as input (FASTQ format), together with associated clinical data (i.e. the patient diagnosis, age, sex, ethnicity). The genome analysis pipeline aligns the sequence data with the ancestry specific reference genomes. The generated BAM files are then processed through the respective algorithm for variant calling which can detect gene fusion, indels and single nucleotide variants (SNV). Tumour- and germline-specific genomic alterations are then mapped to unique reference proteins using Ensembl DB homo_sapiens_core and UniProt. The system determines the longest, best protein isoform as reference sequence for mapping to the information in the proprietary Nucleus knowledgebase. Details on the NGS validation have been published in the supplement of a previous study.[3]

Evidence mining

The next step of the analytical workflow is to automatically identify comprehensive published knowledge about the clinical implications of a gene alterations. To this end, the system screens all genotype information against the reference information on genes, pathways, biological pathways, variants, treatments, clinical trials etc. in the proprietary Nucleus Data Warehouse. At the core of this repository is a manually curated database of biomarker information, the so-called Drug Response Database (DRDB). The biomedical curation team uses text data mining algorithms and manually classifies pharmacogenomic biomarkers according to three levels of clinical validity (see: 2. List of clinical validity levels).

The validity level is reported directly in the MH Guide report to indicate how clinically actionable a pharmacogenomic biomarker finding might be for an individual patient. Other essential information captured during the curation process is: (i) The variant – i.e. the type of genomic aberration (e.g. SNP, Insertion or Deletion etc.); (ii) the drug or treatment used; (iii) the effect of the variant on treatment - i.e. response, resistance or toxicity; (iv) the quantity of effect – e.g. strong, medium, weak; (v) The observation context (i.e. the disease/disease stage or model system); (vi) a link to the source information and a grading of its reliability.

The DRDB database includes information about any form of genomic aberration including Single Nucleotide Variants (SNVs), Copy Number Variations (CNVs), Fusion Proteins (FPs), Insertions and deletions (InsDels), and combinations thereof. The lineage of the mutation is also captured, for example, whether it is (extrapolated to be) a germline or somatic mutation. Similarly, the database includes information about the drug or treatment associated with a genomic aberration, as well as the source of the information e.g. seen in model systems or patients, and includes MeSH terms and other hierarchical classifications. Variants were matched against mutations logged in the Human gene mutation database (HGMD®Professional) (www.biobase-international.com/hgmd) from BIOBASE Corporation (HGMD; http://www.hgmd.org).[4] The information contained within the DRDB patient and/or tumour mutation profile serves to determine a patient's likelihood of response to therapy, likelihood of resistance to therapy, and likelihood of toxicity.

Clinical Interpretation:

MH Guide provides analytical results and access to biomedical resources for a reliable evidence-based clinical interpretation of the genetic alterations via a web-based user interface from which static reports can be generated. The online report displays the genetic alterations detected in the tumour genome and the potential effects of these alterations on (i) drug efficacy i.e. whether the detected genotype confers likelihood of response or resistance to cancer drug

treatments and (ii) drug toxicity i.e. increased likelihood that the patient might experience adverse drug effects. PharmGKB, another drug-drug interaction and pharmacogenetic database based on the FDA's adverse event reporting system was used for cross-reference and validation.[5,6] PharmGKB's prediction of the drugs most likely to cause adverse drug reactions to the patients was compared to the dataome.

2. MH Guide Biomarker validity scores

In general, a biomarker is any characteristic that is objectively measured and evaluated as an indicator of normal biological processes, pathological processes, or pharmacological response to a therapeutic intervention. In the context of the MH Guide, reported biomarkers predict a patient's response to therapy and are based on the characterization of the patient/tumor genomic DNA. Depending on the analysis type, such genomic characteristics can include single nucleotide variants (SNVs), insertions and deletions (indels), fusion genes, and copy number alterations (CNAs).

Biomarker validity:
Results of our proprietary meta-analysis of a patient's biomarkers as an indication of how well validated the biomarker is in its ability to predict response to the associated therapy. There are three validity levels as represented below:

Clinically approved (3) - A companion diagnostic test for this biomarker has been approved by a regulatory agency such as the FDA to predict a specific effect of the drug (i.e., response, resistance, or toxicity) in the patient's disease or tumor type.

Clinical (2) - A biomarker test has not yet been approved by a regulatory agency for the patient's disease but this biomarker has been observed in patients to
predict a specific effect of the drug (i.e., response, resistance, or toxicity) in other diseases or conditions.

Pre-clinical (1) – The biomarker has not yet been observed/tested in patients to predict a specific effect of the drug. Such biomarkers have however been observed either a) in preclinical contexts (e.g., cell lines) or b) predicted by computational method and/or expert-level analysis.

3. List of targeted genes

ABCB1
ABCC1
ABCC2
ABCC6
ABCG2
ABL1
ABL2
ACE
ACPP
ACVRL1
ADA
ADAM15
AFP
AKT1
AKT2
AKT3
ALK
ALOX12
ALOX12B
ANGPT1
ANGPT2
APC
AR
ARAF
ARFRP1
ARID1A
ARID2
ASXL1
ATM
ATP1A3
ATP7A
ATR
ATRX
AURKA
AURKB
AURKC
AVPR2
AXL
BAD
BAP1
BARD1
BBC3
BCL2
BCL2A1
BCL2L1
BCL2L2
BCL6
BCOR
BCORL1
BCR
BGLAP
BIRC2
BIRC3
BIRC5
BLM
BMI1
BMP10
BRAF
BRCA1
BRCA2
BRIP1
BTK
BUB1
CA9
CALCA
CALCR
CALM1
CALM2
CALM3
CARD11
CASP3
CASP7
CASP8
CASP9
CBFB
CBL
CBR3
CCL3
CCND1
CCND2
CCND3
CCNE1
CCNG1
CCR4
CCR5
CD109
CD151
CD19
CD22
CD248
CD274
CD37
CD38
CD4
CD40
CD44
CD52
CD70
CD74
CD79A
CD79B
CDA
CDC7
CDC73
CDH1
CDH2
CDH20
CDH5
CDK1
CDK12
CDK2
CDK4
CDK6
CDK7
CDK8
CDK9
CDKN1B
CDKN2A
CDKN2B
CDKN2C
CDKN2D
CEACAM5
CEBPA
CENPE
CHEK1
CHEK2
CIAPIN1
CIC
CLDN18
CLU
CREBBP
CRKL
CRLF2
CSF1R
CSF2
CTCF
CTLA4
CTNNA1
CTNNB1
CTSG
CXCL12
CXCR1
CXCR2
CYBA
CYP11B1
CYP11B2
CYP17A1
CYP19A1
CYP1B1
CYP2C19
CYP2C8
CYP2C9
CYP2D6
CYP3A4
CYP4B1
DAXX
DCT
DDR2
DDX5
DHFR
DIABLO
DKK1
DLL4
DNMT3A
DOT1L
DPP4
DPYD
E2F1
EDNRA
EDNRB
EEF2
EGFL7
EGFR
EIF4EBP1
EML4
EMSY
ENG
EP300
EPCAM
EPHA3
EPHA5
EPHA6
EPHA7
EPHB1
EPHB4
EPHB6
ERBB2
ERBB3
ERBB4
ERCC2
ERCC5
ERG
ESR1
ESR2
ETV1
ETV4
ETV5
ETV6
EWSR1
EZH2
F13B
F2
F3
F5
FAM123B
FAM46C
FANCA
FANCC
FANCD2
FANCE
FANCF
FANCG
FANCL
FAS
FASLG
FBXW7
FGF1
FGF10
FGF14
FGF19
FGF2
FGF23
FGF3
FGF4
FGF6
FGFR1
FGFR2
FGFR3
FGFR4
FLT1
FLT3
FLT3LG
FLT4
FOLH1
FOLR1
FOXL2
FOXP4
FYN
FZD1
FZD10
FZD2
FZD5
FZD7
FZD8
G6PD
GATA1
GATA2
GATA3
GATA4
GDF2
GGH
GID4
GLP2R
GNA11
GNA13
GNAQ
GNAS
GNRHR
GPC3
GPR124
GRIN2A
GRIN3B
GRM3
GSK3B
GSTO1
GSTO2
GSTP1
GUCY1A2
GUSB
HBB
HBEGF
HDAC1
HDAC11
HDAC2
HDAC6
HFE
HGF
HIF1A
HOXA3
HPSE
HRAS
HRH2
HSP90AA1
HSP90AB1
HSP90B1

HSPA5
IDH1
IDH2
IFNA2
IFNB1
IFNG
IGF1R
IGF2R
IKBKE
IKZF1
IL11RA
IL13
IL13RA2
IL1A
IL2
IL21R
IL25
IL29
IL2RA
IL4
IL4R
IL7R
INHBA
INSR
IRF4
IRS2
ITGA1
ITGA5
ITGAM
ITGAV
ITGB1
ITGB2
ITGB3
ITGB5
ITGB6
ITK
ITPA
JAK1
JAK2
JAK3
JUN
KDM5A
KDM5C
KDM6A
KDR
KEAP1
KIF11
KIR2DL1
KIT
KLF4
KLHL6
KLK2
KLK3
KRAS
LAG3
LGALS1
LHCGR
LOXL2
LPA
LRP1B
LRP2
LRP6
LTA
LTF
LTK
LYN
MAGEA1
MAGEA4
MAP2K1
MAP2K2
MAP2K4
MAP3K1
MAPK1
MAPK3
MCL1
MDM2
MDM4
MED1
MED12
MEF2B
MEFV
MEN1
MET
MGMT
MITF
MLH1
MLL
MLL2
MMP2
MPL
MRE11A
MS4A1
MSH2
MSH3
MSH6
MST1R
MSTN
MTF1
MTHFR
MTOR
MTR
MUTYH
MYC
MYCL1
MYCN
MYD88
MYH11
MYST3
NAE1
NAMPT
NAT2
NCF2
NCL
NF1
NF2
NFE2L2
NFKB1
NFKBIA
NGF
NKX2-1
NOD2
NOTCH1
NOTCH2
NOTCH3
NPM1
NQO1
NR4A1
NRAS
NRP2
NTRK1
NTRK2
NTRK3
NUP93
OPRD1
P2RX7
PAK3
PALB2
PARP1
PARP2
PARP8
PAX5
PBRM1
PDCD1
PDCD1LG2
PDGFB
PDGFRA
PDGFRB
PDK1
PDPK1
PGF
PGR
PHLPP2
PIK3CA
PIK3CB
PIK3CD
PIK3CG
PIK3R1
PIK3R2
PIM1
PLA2G10
PLA2G12A
PLA2G12B
PLA2G1B
PLA2G2A
PLA2G2D
PLA2G2E
PLA2G2F
PLA2G3
PLA2G5
PLA2G6
PLAU
PLCG1
PLK1
PLK4
PMP22
PNP
POLD1
POLE
PPARA
PPARD
PPARG
PPP2R1A
PRAME
PRDM1
PRKAR1A
PRKCA
PRKCB
PRKDC
PRLR
PRSS1
PSMB5
PSMB8
PTCH1
PTCH2
PTEN
PTGS2
PTH
PTK2B
PTPN11
PTPRC
PTPRD
RAD50
RAD51
RAD51L3
RAF1
RARA
RB1
RET
RHEB
RICTOR
RNF43
ROCK2
ROS1
RPE65
RPS27A
RPTOR
RRM2
RUNX1
RUNX3
S100A9
S1PR1
SELL
SELP
SERPINA1
SERPINE1
SETD2
SF3B1
SFTPC
SHH
SLC10A3
SLC16A1
SLC19A1
SLC29A1
SLC5A5
SLC6A2
SLC7A11
SLCO1B1
SMAD2
SMAD3
SMAD4
SMARCA4
SMARCB1
SMC4
SMO
SOCS1
SOD2
SOX10
SOX2
SPEN
SPG7
SPOP
SPP1
SRC
SSTR1
SSTR2
SSTR3
SSTR4
SSTR5
STAG2
STAT3
STAT4
STK11
SUFU
SULT1C4
SYK
T
TACR1
TBX22
TEC
TEK
TERT
TET1
TET2
TGFB1
TGFBR1
TGFBR2
TGM2
TH
TLR2
TLR3
TLR4
TLR5
TLR7
TLR8
TLR9
TMPRSS2
TNC
TNF
TNFAIP3
TNFRSF10A
TNFRSF10B
TNFRSF14
TNFRSF4
TNFRSF8

TNFRSF9	TP53	TUSC2	UMPS	VPS4B	XRCC2
TNFSF10	TP63	TYMS	USP9X	VWF	YES1
TNFSF13	TP73	TYR	VCAM1	WISP3	ZEB2
TNFSF13B	TPMT	UBA52	VDR	WT1	ZNF217
TNKS	TRPM8	UBB	VEGFB	XIAP	ZNF703
TOP1	TSC1	UBC	VEGFC	XPC	
TOP2A	TSC2	UGT1A1	VHL	XPO1	
TOR1A	TSHR	UGT1A7	VKORC1	XRCC1	

4. References

1. Bohnert R, Vivas S, Jansen G. Comprehensive benchmarking of SNV callers for highly admixed tumor data. *PloS One*. 2017;12(10):e0186175. doi:10.1371/journal.pone.0186175

2. Rieber N, Bohnert R, Ziehm U, Jansen G. Reliability of algorithmic somatic copy number alteration detection from targeted capture data. *Bioinformatics*. 2017;33(18):2791-2798. doi:10.1093/bioinformatics/btx284

3. Malgerud L, Lindberg J, Wirta V, et al. Bioinformatory-assisted analysis of next-generation sequencing data for precision medicine in pancreatic cancer. *Mol Oncol*. 2017;11(10):1413-1429. doi:10.1002/1878-0261.12108

4. Stenson PD, Mort M, Ball EV, et al. The Human Gene Mutation Database: 2008 update. *Genome Med*. 2009;1(1):13. doi:10.1186/gm13

5. Whirl-Carrillo M, McDonagh EM, Hebert JM, et al. Pharmacogenomics knowledge for personalized medicine. *Clin Pharmacol Ther*. 2012;92(4):414-417. doi:10.1038/clpt.2012.96

6. Thorn CF, Klein TE, Altman RB. Pharmacogenomics and bioinformatics: PharmGKB. *Pharmacogenomics*. 2010;11(4):501-505. doi:10.2217/pgs.10.15
