# Supplementary material for: Consistency of a clinical decision support system with molecular tumour board recommendations for tumour sequencing-guided treatment of pancreatic cancer
Source: ESMO Gastrointest Oncol. 2024 Jun 19;5:100070. doi: 10.1016/j.esmogo.2024.100070 (PMC12836574; doi:10.1016/j.esmogo.2024.100070)
Supplement: Supplementary Table S3 [file mmc4.pdf]

Consistency of a clinical decision support system with molecular tumour board recommendations for tumour sequencing-guidedtreatment of pancreatic cancer: A prospective observational study

Supplemental Table 3

Gene	Variant			Assertion	CDSS reference (PMID)*	CDSS grade	Emulated ESCAT score†
KRAS	*G12R*	*G12V*	*G12D*	Dactolisib and MEK inhibitor	25344362, 26663013, 24915778	1 “Pre-clinical”	IV-A
	G12R	*G12D*		Sorafenib	22282465, 23177514, 23404247, 24024839, 25313136		References not relevant for assertion / no tier assigned
	G12V	*G12A*					
				MEK inhibitors			II-A
TP53	*Y234C*	*R333fs*	*H193L*	PARP inhibitors	25733866, 22446188, 26312527, 22101337	1 “Pre-clinical”	IV-A
	D259V	*L111P*	*P152fs*				
	R273H	*H193R*	*Y220S*				
	R273C	*M246K*	*S127F*				
	*R213**	*C238F*					
	L206fs	*C275fs*					
CDKN2A	*W110**			CDK inhibitors	24369047, 24795392, 26324739	3 “Clinical approved”	References not relevant for assertion / no tier assigned
	T79fs			CDK inhibitors	24369047, 24795392, 24495407, 26324739, 25156567	2 “Clinical”	IV-A
	V126D			CDK inhibitors	21462282, 24495407, 26324739, 19260062, 20340136	1 “Pre-clinical”	IV-A
	Y44fs			CDK inhibitors	24369047, 24795392, 24495407, 26324739, 25156567	2 “Clinical”	IV-A
	A60fs			CDK inhibitors	24495407, 25156567, 26380006, 27196769, 27542767	1 “Pre-clinical”	III-A
	E69 and E61fs*						
	L78fs			CDK inhibitors	24495407, 26380006, 27030077, 27196769, 27542767	1 “Pre-clinical”	III-A
	*Y129**			CDK inhibitors	24369047, 24795392, 24495407, 26324739, 25156567	1 “Pre-clinical”	IV-A
ATM	*A2301fs*	*N314fs*	*L2656fs*	PARP inhibitors	23272087, 24252502, 21034966	1 “Pre-clinical”	IV-A
SMAD4	*S178*	*S357fs*		Everolimus<br>MEK inhibitor ± RAF inhibotor	12010891, 19584151, 22860091, 23443316, 23470568, 24025354, 25760429	1 “Pre-clinical”	X
STK11	*K84**	*E57fs*		Everolimus	24218567, 12097271, 21189378, 15863673	1 “Pre-clinical”	II-B
BRCA1	*L1209fs*			PARP inhibitors	26180927, 26399274, 25757679, 21183737, 27002934	2 “Clinical”	II-B
BRCA2	*K434fs*			PARP inhibitors	21183737, 25757679, 26180927, 26399274, 27002934	2 “Clinical”	II-B
CTNNB1	*S45F*			Imatinib	18832571, 21478276, 23913621, 23960186, 24788118, 25341748, 25838078, 26861905, 28350521	2 “Clinical”	X
ERBB2	*D769Y*			Neratinib, Lapatinib	22046346, 23220880	1 “Pre-clinical”	IV-A
RET	*Y791F*			Lenvatinib, Vandetanib, Cabozantinib, Sorafenib	20065189, 24561444	1 “Pre-clinical”	III-A
MLH1	*I219V*			Pembrolizumab	21642682, 24944470, 26028255, 16166421	2 “Clinical”	X

*A complete reference list of assertions is available below; †Emulated score inferred from assertions made by CDSS

KRAS
PMID: **25344362**[1]
PMID: **26663013**[2]
PMID: **24915778**[3]

PMID: **22282465**[4]
PMID: **23177514**[5]
PMID: **23404247**[6]
PMID: **24024839**[7]
PMID: **25313136**[8]

TP53
PMID: **25733866**[9]
PMID: **22446188**[10]
PMID: **26312527**[11]
PMID: **22101337**[12]

CDKN2A
PMID: **24369047**[13]
PMID: **24795392**[14]
PMID: **26324739**[15]
PMID: **24495407**[16]
PMID: **25156567**[17]
PMID: **21462282**[18]
PMID: **19260062**[19]
PMID: **20340136**[20]
PMID: **26380006**[21]
PMID: **27196769**[22]
PMID: **27542767**[23]
PMID: **27030077**[24]

ATM
PMID: **24252502**[25]
PMID: **21034966**[26]
PMID: **23272087**[27]

SMAD4
PMID: **12010891**[28]
PMID: **19584151**[29]
PMID: **22860091**[30]
PMID: **23443316**[31]
PMID: **23470568**[32]
PMID: **24025354**[33]
PMID: **25760429**[34]

STK11
PMID: **24218567**[35]
PMID: **12097271**[36]
PMID: **21189378**[37]
PMID: **15863673**[38]

BRCA1
PMID: **26180927**[39]
PMID: **26399274**[40]
PMID: **25757679**[41]
PMID: **21183737**[42]
PMID: **27002934**[43]

BRCA2
PMID: **21183737**[42]
PMID: **25757679**[41]
PMID: **26180927**[39]
PMID: **26399274**[40]
PMID: **27002934**[43]

CTNNB1
PMID: **18832571**[44]
PMID: **21478276**[45]
PMID: **23913621**[46]
PMID: **23960186**[47]
PMID: **24788118**[48]
PMID: **25341748**[49]
PMID: **25838078**[50]
PMID: **26861905**[51]
PMID: **28350521**[52]

ERBB2
PMID: **22046346**[53]
PMID: **23220880**[54]

RET
PMID: **20065189**[55]
PMID: **24561444**[56]

MLH1
PMID: **21642682**[57]
PMID: **24944470**[58]
PMID: **26028255**[59]
PMID: **16166421**[60]

1. Tolcher AW, Bendell JC, Papadopoulos KP, et al. A phase IB trial of the oral MEK inhibitor trametinib (GSK1120212) in combination with everolimus in patients with advanced solid tumors. *Ann Oncol*. 2015;26(1):58-64. doi:10.1093/annonc/mdu482
2. Witteck L, Jaster R. Trametinib and dactolisib but not regorafenib exert antiproliferative effects on rat pancreatic stellate cells. *Hepatobiliary Pancreat Dis Int*. 2015;14(6):642-650. doi:10.1016/s1499-3872(15)60032-7
3. Infante JR, Somer BG, Park JO, et al. A randomised, double-blind, placebo-controlled trial of trametinib, an oral MEK inhibitor, in combination with gemcitabine for patients with untreated metastatic adenocarcinoma of the pancreas. *Eur J Cancer*. 2014;50(12):2072-2081. doi:10.1016/j.ejca.2014.04.024
4. Miranda C, Nucifora M, Molinari F, et al. KRAS and BRAF mutations predict primary resistance to imatinib in gastrointestinal stromal tumors. *Clin Cancer*. 2012;18(6):1769-1776. doi:10.1158/1078-0432.CCR-11-2230
5. Grothey A, Van Cutsem E, Sobrero A, et al. Regorafenib monotherapy for previously treated metastatic colorectal cancer (CORRECT): an international,

multicentre, randomised, placebo-controlled, phase 3 trial. *Lancet*. 2013;381(9863):303-312. doi:10.1016/S0140-6736(12)61900-X
6. Valtorta E, Misale S, Sartore-Bianchi A, et al. KRAS gene amplification in colorectal cancer and impact on response to EGFR-targeted therapy. *Int J Cancer*. 2013;133(5):1259-1265. doi:10.1002/ijc.28106
7. Douillard JY, Oliner KS, Siena S, et al. Panitumumab-FOLFOX4 treatment and RAS mutations in colorectal cancer. *N Engl J Med*. 2013;369(11):1023-1034. doi:10.1056/NEJMoa1305275
8. Said R, Ye Y, Falchook GS, et al. Outcomes of patients with advanced cancer and KRAS mutations in phase I clinical trials. *Oncotarget*. 2014;5(19):8937-8946. doi:10.18632/oncotarget.2339
9. Polotskaia A, Xiao G, Reynoso K, et al. Proteome-wide analysis of mutant p53 targets in breast cancer identifies new levels of gain-of-function that influence PARP, PCNA, and MCM4. *Proc Natl Acad Sci U S A*. 2015;112(11):E1220-1229. doi:10.1073/pnas.1416318112
10. Ma CX, Cai S, Li S, et al. Targeting Chk1 in p53-deficient triple-negative breast cancer is therapeutically beneficial in human-in-mouse tumor models. *J Clin Invest*. 2012;122(4):1541-1552. doi:10.1172/JCI58765
11. Dale Rein I, Solberg Landsverk K, Micci F, Patzke S, Stokke T. Replication-induced DNA damage after PARP inhibition causes G2 delay, and cell line-dependent apoptosis, necrosis and multinucleation. *Cell Cycle*. 2015;14(20):3248-3260. doi:10.1080/15384101.2015.1085137
12. Nguyen D, Zajac-Kaye M, Rubinstein L, et al. Poly(ADP-ribose) polymerase inhibition enhances p53-dependent and -independent DNA damage responses induced by DNA damaging agent. *Cell Cycle*. 2011;10(23):4074-4082. doi:10.4161/cc.10.23.18170
13. Rocca A, Farolfi A, Bravaccini S, Schirone A, Amadori D. Palbociclib (PD 0332991) : targeting the cell cycle machinery in breast cancer. *Expert Opin Pharmacother*. 2014;15(3):407-420. doi:10.1517/14656566.2014.870555
14. Dickson MA. Molecular pathways: CDK4 inhibitors for cancer therapy. *Clin Cancer Res*. 2014;20(13):3379-3383. doi:10.1158/1078-0432.CCR-13-1551
15. Beaver JA, Amiri-Kordestani L, Charlab R, et al. FDA Approval: Palbociclib for the treatment of postmenopausal patients with estrogen receptor-positive, HER2-negative metastatic breast cancer. *Clin Cancer Res*. 2015;21(21):4760-4766. doi:10.1158/1078-0432.CCR-15-1185
16. Young RJ, Waldeck K, Martin C, et al. Loss of CDKN2A expression is a frequent event in primary invasive melanoma and correlates with sensitivity to the CDK4/6 inhibitor PD0332991 in melanoma cell lines. *Pigment Cell Melanoma Res*. 2014;27(4):590-600. doi:10.1111/pcmr.12228
17. Franco J, Witkiewicz AK, Knudsen ES. CDK4/6 inhibitors have potent activity in combination with pathway selective therapeutic agents in models of pancreatic cancer. *Oncotarget*. 2014;5(15):6512-6525. doi:10.18632/oncotarget.2270
18. Miller PJ, Duraisamy S, Newell JA, et al. Classifying variants of CDKN2A using computational and laboratory studies. *Hum Mutat*. 2011;32(8):900-911. doi:10.1002/humu.21504

19. Kannengiesser C, Brookes S, del Arroyo AG, et al. Functional, structural, and genetic evaluation of 20 CDKN2A germ line mutations identified in melanoma-prone families or patients. *Hum Mutat*. 2009;30(4):564-574. doi:10.1002/humu.20845
20. McKenzie HA, Fung C, Becker TM, et al. Predicting functional significance of cancer-associated p16(INK4a) mutations in CDKN2A. *Hum Mutat*. 2010;31(6):692-701. doi:10.1002/humu.21245
21. Huang S, Ye H, Guo W, et al. CDK4/6 inhibitor suppresses gastric cancer with CDKN2A mutation. *Int J Clin Exp Med*. 2015;8(7):11692-11700.
22. Helsten T, Kato S, Schwaederle M, et al. Cell-cycle gene alterations in 4,864 tumors analyzed by Next-Generation Sequencing: Implications for targeted therapeutics. *Mol Cancer Ther*. 2016;15(7):1682-1690. doi:10.1158/1535-7163.MCT-16-0071
23. Infante JR, Cassier PA, Gerecitano JF, et al. A phase I study of the cyclin-dependent kinase 4/6 inhibitor ribociclib (LEE011) in patients with advanced solid tumors and lymphomas. *Clin Cancer Res*. 2016;22(23):5696-5705. doi:10.1158/1078-0432.CCR-16-1248
24. O'Leary B, Finn RS, Turner NC. Treating cancer with selective CDK4/6 inhibitors. *Nat Rev Clin Oncol*. 2016;13(7):417-430. doi:10.1038/nrclinonc.2016.26
25. Gilardini Montani MS, Prodosmo A, Stagni V, et al. ATM-depletion in breast cancer cells confers sensitivity to PARP inhibition. *J Exp Clin Cancer Res*. 2013;32(1):95. doi:10.1186/1756-9966-32-95
26. Smith J, Tho LM, Xu N, Gillespie DA. The ATM-Chk2 and ATR-Chk1 pathways in DNA damage signaling and cancer. *Adv Cancer Res*. 2010;108:73-112. doi:10.1016/B978-0-12-380888-2.00003-0
27. Chiu YT, Liu J, Tang K, Wong YC, Khanna KK, Ling MT. Inactivation of ATM/ATR DNA damage checkpoint promotes androgen induced chromosomal instability in prostate epithelial cells. *PloS One*. 2012;7(12):e51108. doi:10.1371/journal.pone.0051108
28. Biankin AV, Biankin SA, Kench JG, et al. Aberrant p16(INK4A) and DPC4/Smad4 expression in intraductal papillary mucinous tumours of the pancreas is associated with invasive ductal adenocarcinoma. *Gut*. 2002;50(6):861-868. doi:10.1136/gut.50.6.861
29. Blackford A, Serrano OK, Wolfgang CL, et al. SMAD4 gene mutations are associated with poor prognosis in pancreatic cancer. *Clin Cancer Res*. 2009;15(14):4674-4679. doi:10.1158/1078-0432.CCR-09-0227
30. Jiang H, He C, Geng S, et al. RhoT1 and Smad4 are correlated with lymph node metastasis and overall survival in pancreatic cancer. *PloS One*. 2012;7(7):e42234. doi:10.1371/journal.pone.0042234
31. Le Gendre O, Sookdeo A, Duliepre SA, Utter M, Frias M, Foster DA. Suppression of AKT phosphorylation restores rapamycin-based synthetic lethality in SMAD4-defective pancreatic cancer cells. *Mol Cancer Res*. 2013;11(5):474-481. doi:10.1158/1541-7786.MCR-12-0679
32. Oshima M, Okano K, Muraki S, et al. Immunohistochemically detected expression of 3 major genes (CDKN2A/p16, TP53, and SMAD4/DPC4) strongly predicts survival in patients with resectable pancreatic cancer. *Ann Surg*. 2013;258(2):336-346. doi:10.1097/SLA.0b013e3182827a65

33. Ai X, Wu Y, Zhang W, et al. Targeting the ERK pathway reduces liver metastasis of Smad4-inactivated colorectal cancer. *Cancer Biol Ther*. 2013;14(11):1059-1067. doi:10.4161/cbt.26427
34. Yamada S, Fujii T, Shimoyama Y, et al. SMAD4 expression predicts local spread and treatment failure in resected pancreatic cancer. *Pancreas*. 2015;44(4):660-664. doi:10.1097/MPA.0000000000000315
35. Lai LP, Lilley BN, Sanes JR, McMahon AP. Lkb1/Stk11 regulation of mTOR signaling controls the transition of chondrocyte fates and suppresses skeletal tumor formation. *Proc Natl Acad Sci U S A*. 2013;110(48):19450-19455. doi:10.1073/pnas.1309001110
36. Sanchez-Cespedes M, Parrella P, Esteller M, et al. Inactivation of LKB1/STK11 is a common event in adenocarcinomas of the lung. *Cancer Res*. 2002;62(13):3659-3662.
37. Klümpen HJ, Queiroz KCS, Spek CA, et al. mTOR Inhibitor Treatment of Pancreatic Cancer in a Patient With Peutz-Jeghers Syndrome. *J Clin Oncol*. 2011;29(6):e150-e153. doi:10.1200/JCO.2010.32.7825
38. Schumacher V, Vogel T, Leube B, et al. STK11 genotyping and cancer risk in Peutz-Jeghers syndrome. *J Med Genet*. 2005;42(5):428-435. doi:10.1136/jmg.2004.026294
39. van der Noll R, Marchetti S, Steeghs N, et al. Long-term safety and anti-tumour activity of olaparib monotherapy after combination with carboplatin and paclitaxel in patients with advanced breast, ovarian or fallopian tube cancer. *Br J Cancer*. 2015;113(3):396-402. doi:10.1038/bjc.2015.256
40. Bao Z, Cao C, Geng X, et al. Effectiveness and safety of poly (ADP-ribose) polymerase inhibitors in cancer therapy: A systematic review and meta-analysis. *Oncotarget*. 2016;7(7):7629-7639. doi:10.18632/oncotarget.5367
41. Gunderson CC, Moore KN. Olaparib: an oral PARP-1 and PARP-2 inhibitor with promising activity in ovarian cancer. *Future Oncol*. 2015;11(5):747-757. doi:10.2217/fon.14.313
42. Drew Y, Mulligan EA, Vong WT, et al. Therapeutic potential of poly(ADP-ribose) polymerase inhibitor AG014699 in human cancers with mutated or methylated BRCA1 or BRCA2. *J Natl Cancer Inst*. 2011;103(4):334-346. doi:10.1093/jnci/djq509
43. Drew Y, Ledermann J, Hall G, et al. Phase 2 multicentre trial investigating intermittent and continuous dosing schedules of the poly(ADP-ribose) polymerase inhibitor rucaparib in germline BRCA mutation carriers with advanced ovarian and breast cancer. *Br J Cancer*. 2016;114(7):723-730. doi:10.1038/bjc.2016.41
44. Lazar AJF, Tuvin D, Hajibashi S, et al. Specific mutations in the beta-catenin gene (CTNNB1) correlate with local recurrence in sporadic desmoid tumors. *Am J Pathol*. 2008;173(5):1518-1527. doi:10.2353/ajpath.2008.080475
45. Kasper B, Ströbel P, Hohenberger P. Desmoid tumors: clinical features and treatment options for advanced disease. *Oncologist*. 2011;16(5):682-693. doi:10.1634/theoncologist.2010-0281
46. Colombo C, Miceli R, Lazar AJ, et al. CTNNB1 45F mutation is a molecular prognosticator of increased postoperative primary desmoid tumor recurrence: an independent, multicenter validation study. *Cancer*. 2013;119(20):3696-3702. doi:10.1002/cncr.28271

47. Mullen JT, DeLaney TF, Rosenberg AE, et al. β-Catenin mutation status and outcomes in sporadic desmoid tumors. *Oncologist*. 2013;18(9):1043-1049. doi:10.1634/theoncologist.2012-0449
48. Hamada S, Futamura N, Ikuta K, et al. CTNNB1 S45F mutation predicts poor efficacy of meloxicam treatment for desmoid tumors: a pilot study. *PloS One*. 2014;9(5):e96391. doi:10.1371/journal.pone.0096391
49. van Broekhoven DLM, Verhoef C, Grünhagen DJ, et al. Prognostic value of CTNNB1 gene mutation in primary sporadic aggressive fibromatosis. *Ann Surg Oncol*. 2015;22(5):1464-1470. doi:10.1245/s10434-014-4156-x
50. Aitken SJ, Presneau N, Kalimuthu S, et al. Next-generation sequencing is highly sensitive for the detection of beta-catenin mutations in desmoid-type fibromatoses. *Virchows Arch*. 2015;467(2):203-210. doi:10.1007/s00428-015-1765-0
51. Kasper B, Gruenwald V, Reichardt P, Bauer S, Hohenberger P, Haller F. Correlation of CTNNB1 mutation status with progression arrest rate in RECIST progressive desmoid-type fibromatosis treated with imatinib: Translational research results from a phase 2 study of the German Interdisciplinary Sarcoma Group (GISG-01). *Ann Surg Oncol*. 2016;23(6):1924-1927. doi:10.1245/s10434-016-5132-4
52. Kummar S, O'Sullivan Coyne G, Do KT, et al. Clinical activity of the γ-secretase inhibitor PF-03084014 in adults with desmoid tumors (aggressive fibromatosis). *J Clin Oncol*. 2017;35(14):1561-1569. doi:10.1200/JCO.2016.71.1994
53. Kancha RK, von Bubnoff N, Bartosch N, Peschel C, Engh RA, Duyster J. Differential sensitivity of ERBB2 kinase domain mutations towards lapatinib. *PloS One*. 2011;6(10):e26760. doi:10.1371/journal.pone.0026760
54. Bose R, Kavuri SM, Searleman AC, et al. Activating HER2 mutations in HER2 gene amplification negative breast cancer. *Cancer Discov*. 2013;3(2):224-237. doi:10.1158/2159-8290.CD-12-0349
55. Wells SA, Gosnell JE, Gagel RF, et al. Vandetanib for the treatment of patients with locally advanced or metastatic hereditary medullary thyroid cancer. *J Clin Oncol*. 2010;28(5):767-772. doi:10.1200/JCO.2009.23.6604
56. Mulligan LM. RET revisited: expanding the oncogenic portfolio. *Nat Rev Cancer*. 2014;14(3):173-186. doi:10.1038/nrc3680
57. Bonadona V, Bonaïti B, Olschwang S, et al. Cancer risks associated with germline mutations in MLH1, MSH2, and MSH6 genes in Lynch syndrome. *JAMA*. 2011;305(22):2304-2310. doi:10.1001/jama.2011.743
58. Saridaki Z, Souglakos J, Georgoulias V. Prognostic and predictive significance of MSI in stages II/III colon cancer. *World J Gastroenterol*. 2014;20(22):6809-6814. doi:10.3748/wjg.v20.i22.6809
59. Le DT, Uram JN, Wang H, et al. PD-1 Blockade in Tumors with Mismatch-Repair Deficiency. *N Engl J Med*. 2015;372(26):2509-2520. doi:10.1056/NEJMoa1500596
60. Gill S, Lindor NM, Burgart LJ, et al. Isolated loss of PMS2 expression in colorectal cancers: frequency, patient age, and familial aggregation. *Clin Cancer Res*. 2005;11(18):6466-6471. doi:10.1158/1078-0432.CCR-05-0661
