# Supplementary material for: Consistency of a clinical decision support system with molecular tumour board recommendations for tumour sequencing-guided treatment of pancreatic cancer
Source: ESMO Gastrointest Oncol. 2024 Jun 19;5:100070. doi: 10.1016/j.esmogo.2024.100070 (PMC12836574; doi:10.1016/j.esmogo.2024.100070)
Supplement: Supplementary Table S4 [file mmc5.pdf]

Consistency of a clinical decision support system with molecular tumour board recommendations for tumour sequencing-guided treatment of pancreatic cancer: A prospective observational study

Supplemental Table 3

Reported variants flagged as inefficacy markers for cancer drugs

Patient	Drug	Biomarker	Validity level
103	Everolimus	KRAS.G12R	2
	Trametinib		2
106	Everolimus	KRAS.G12R	2
	Trametinib		2
107	Everolimus	KRAS.G12V	2
	Trametinib		2
108	Trametinib	KRAS.G12D	2
			2
109	Tamoxifen	CYP2D6.P34S	2
110	Everolimus	KRAS.G12R	2
	MEK inhibitors		2
	Tamoxifen	CYP2D6.P34S	2
112	Everolimus	KRAS.G12D	2
	Trametinib		2
113	Tamoxifen	CYP2D6.P34S	2
114	EGFR inhibitors	KRAS.G12R	2
117	Tamoxifen	CYP2D6.P34S	2
	EGFR inhibitors	KRAS.G12V	2
118	Meloxicam	CTNNB1.S45F	2
119	Tamoxifen	CYP2D6.P34S	2
	EGFR inhibitors	KRAS.G12V	2
120	Tamoxifen	CYP2D6.P34S	2
	EGFR inhibitors	KRAS.G12D	2
121	EGFR inhibitors	KRAS.G12V	2
122	EGFR inhibitors	KRAS.G12D	2
124	EGFR inhibitors	KRAS.G12R	2
125	EGFR inhibitors	KRAS.G12V	2
126	EGFR inhibitors	KRAS.G12V	2
127	EGFR inhibitors	KRAS.G12V	2
128	Tamoxifen	CYP2D6.P34S	2
	EGFR inhibitors	KRAS.G12V	2
129	Tamoxifen	CYP2D6.P34S	2
	EGFR inhibitors	KRAS.G12D	2
130	Tamoxifen	CYP2D6.W152fs	2
	EGFR inhibitors	KRAS.G12A	2
131	EGFR inhibitors	KRAS.G12D	2
132	EGFR inhibitors	KRAS.G12D	2
135	EGFR inhibitors	KRAS.G12D	2
136	EGFR inhibitors	KRAS.G12D	2
139	EGFR inhibitors	KRAS.G12V	2

**Summary**	
KRAS.G12D	10
KRAS.G12V	10
KRAS.G12R	8
CYP2D6.P34S	8
CTNNB1.S45F	1
CYP2D6.W152fs	1
KRAS.G12A	1
**Total**	**39**
