# Supplementary material for: Consistency of a clinical decision support system with molecular tumour board recommendations for tumour sequencing-guided treatment of pancreatic cancer
Source: ESMO Gastrointest Oncol. 2024 Jun 19;5:100070. doi: 10.1016/j.esmogo.2024.100070 (PMC12836574; doi:10.1016/j.esmogo.2024.100070)
Supplement: Supplementary Table S5 [file mmc6.pdf]

Consistency of a clinical decision support system with molecular tumour board recommendations for tumour sequencing-guided treatment of pancreatic cancer: A prospective observational study

Supplemental Table 4

Heterozygous germline variants with increased risks of cancer treatment-associated toxicity

Patient	Treatment	Biomarker	Validity level
106	Methotrexate	MTHFR.A222V	2
	Platinum-based agents	XRCC1.R399Q	2
	Cyclophosphamide		2
107	Tamoxifen	F5.R534Q	3
	Methotrexate	MTHFR.A222V	2
108	Platinum-based agents	XRCC1.R399Q	2
	Cyclophosphamide		2
	Radiotherapy	ERCC2.D312N	2
109	Platinum-based agents	XRCC1.R399Q	2
	Cyclophosphamide		2
	Gefitinib	CYP2D6.P34S	1
	Radiotherapy	ERCC2.D312N	2
		ERCC2.K751Q	2
110	Platinum-based agents	XRCC1.R399Q	2
	Cyclophosphamide		2
	Gefitinib	CYP2D6.P34S	1
	Radiotherapy	ERCC2.K751Q	2
111	Methotrexate	MTHFR.A222V	2
	Platinum-based agents	XRCC1.R399Q	2
	Cyclophosphamide		2
	Gemcitabine	CDA.K27Q	2
113	Mercaptopurine	TPMT.Y240C	3
	Thioguanine		3
	Thioguanine	TPMT.A154T	3
	Mercaptopurine		3
	Thioguanine	TPMT.A154T	3
	Tamoxifen	CYP2D6.P34S	2
	Gefitinib		1
	Paclitaxel	CYP2C8.K399R	2
		CYP2C8.R139K	2
	Platinum-based agents	XRCC1.R399Q	2
	Cyclophosphamide		2
	Radiotherapy	ERCC2.D312N	2
		ERCC2.K751Q	2
	Methotrexate	MTHFR.A222V	2
114	Platinum-based agents	XRCC1.R399Q	2
	Cyclophosphamide		2
	Radiotherapy	ERCC2.D312N	2
115	Radiotherapy	ERCC2.K751Q	2

[illegible]	Radiotherapy	ERCC2.D312N	2
117	Paclitaxel	CYP2C8.K399R	2
	Cyclophosphamide	XRCC1.R399Q	2
	Platinum-based agents		2
	Paclitaxel	CYP2C8.R139K	2
	Gefitinib	CYP2D6.P34S	1
	Radiotherapy	ERCC2.D312N	2
		ERCC2.K751Q	2
118	Daunorubicin	G6PD.D282H	3
	Doxorubicin		3
	Carmustine		3
	Cisplatin	MUTYH.V22M	2
	Cyclophosphamide		2
	Platinum-based agents	XRCC1.R399Q	2
	Cyclophosphamide		2
	Rasburicase	G6PD.D282H	3
	Dabrafenib		3
	Methylene Blue		3
119	Methotrexate	MTHFR.A222V	2
	Platinum-based agents	XRCC1.R399Q	2
	Cyclophosphamide		2
	Radiotherapy	ERCC2.D312N	2
		ERCC2.K751Q	2
	Gefitinib	CYP2D6.P34S	1
120	Platinum-based agents	XRCC1.R399Q	2
	Cyclophosphamide		2
	Gefitinib	CYP2D6.P34S	1
121	Methotrexate	MTHFR.A222V	2
	Platinum-based agents	XRCC1.R399Q	2
	Cyclophosphamide		2
	Radiotherapy	ERCC2.D312N	2
		ERCC2.K751Q	2
122	Radiotherapy	ERCC2.D312N	2
		ERCC2.K751Q	2
123	Methotrexate	MTHFR.A222V	2
	Cisplatin	MUTYH.V22M	2
	Cyclophosphamide		2
	Radiotherapy	ERCC2.D312N	2
		ERCC2.K751Q	2
124	Thioguanine	TPMT.Y240C	3
	6-Mercaptopurine		3
	Thioguanine	TPMT.A154T	3
	6-Mercaptopurine		3
125	Tamoxifen	F5.R534Q	3
	Methotrexate	MTHFR.A222V	2
	Sunitinib	ABCG2.Q141K	2
	Platinum-based agents	XRCC1.R399Q	2
	Cyclophosphamide		2

	Cytarabine	CDA.K27Q	2
	Gemcitabine		2
	Radiotherapy	ERCC2.K751Q	2
		ERCC2.D312N	2
126	Methotrexate	MTHFR.A222V	2
127	Thioguanine	TPMT.Y240C	3
	6-Mercaptopurine		3
	6-Mercaptopurine	TPMT.A154T	3
	Thioguanine		3
	Cisplatin	MUTYH.V22M	2
		TPMT.A154T	3
		TPMT.Y240C	3
	Platinum-based agents	XRCC1.R399Q	2
	Sunitinib		2
	Cyclophosphamide		2
	Irinotecan	UGT1A.G7IR	2
	Belinostat	UGT1A.G7IR	2
	Radiotherapy	ERCC2.D312N	2
		ERCC2.K751Q	2
	Cyclophosphamide	MUTYH.V22M	2
128	Platinum-based agents	XRCC1.R399Q	2
	Cyclophosphamide		2
	Gefitinib	CYP2D6.P34S	1
	Radiotherapy	ERCC2.D312N	2
		ERCC2.K751Q	2
129	Fluoropyrimidines	DPYD.S534N	2
	Gefitinib	CYP2D6.P34S	1
	Radiotherapy	ERCC2.D312N	2
		ERCC2.K751Q	2
130	Gefitinib	CYP2D6.W152fs	1
	Cisplatin	MUTYH.V22M	2
	Cyclophosphamide		2
	Platinum-based agents	XRCC1.R399Q	2
	Cyclophosphamide		2
131	Docetaxel	GSTP1.A114V	2
	Methotrexate	MTHFR.A222V	2
	Platinum-based agents	XRCC1.R399Q	2
	Cyclophosphamide		2
	Radiotherapy	ERCC2.D312N	2
		ERCC2.K751Q	2
132	Platinum-based agents	XRCC1.R399Q	2
	Cyclophosphamide		2
	Radiotherapy	ERCC2.K751Q	2
		ERCC2.D312N	2
134	Methotrexate	MTHFR.A222V	2
	Sunitinib	ABCG2.Q141K	2
	Radiotherapy	ERCC2.D312N	2
		ERCC2.K751Q	2

135	Methotrexate	MTHFR.A222V	2
	Gemcitabine	CDA.K27Q	2
	Cytarabine		2
136	Docetaxel	GSTP1.A114V	2
	Methotrexate	MTHFR.A222V	2
	Platinum-based agents	XRCC1.R399Q	2
	Cyclophosphamide		2
	Gemcitabine	CDA.K27Q	2
	Radiotherapy	ERCC2.D312N	2
		ERCC2.K751Q	2
139	Gemcitabine	CDA.K27Q	2
	Cytarabine		2
	Platinum-based agents	XRCC1.R399Q	2
	Cyclophosphamide		2
	5-Fluorouracil	UMPS.G213A	2
	Capecitabine		2
	Tegafur		2

**Summary**	
XRCC1.R399Q	20
ERCC2.D312N	18
MTHFR.A222V	13
CYP2D6.P34S	8
CDA.K27Q	5
TPMT.A154T	5
ABCG2.Q141K	2
CYP2C8.K399R	2
F5.R534Q	2
G6PD.D282H	2
GSTP1.A114V	2
UGT1A.G7IR	2
CYP2D6.W152fs	1
DPYD.S534N	1
UMPS.G213A	1
**Total**	84
